# Efficient Compression of Mass Spectrometry Images via Contrastive Learning-Based Encoding

**DOI:** 10.1021/acs.analchem.4c06913

**Published:** 2025-07-21

**Authors:** Piotr Radziński, Jakub Skrajny, Maurycy Moczulski, Michał A. Ciach, Dirk Valkenborg, Benjamin Balluff, Anna Gambin

**Affiliations:** † Institute of Informatics, 49605 University of Warsaw, Stefana Banacha 2, Warsaw 02-097, Poland; ‡ Department of Applied Biomedical Science, Faculty of Health Sciences, University of Malta, Msida, MSD 2080, Malta; § Interuniversity Institute of Biostatistics and Statistical Bioinformatics, 54496 Hasselt University, Hasselt BE3500, Belgium; ∥ The Maastricht MultiModal Molecular Imaging (M4I) Institute, 5211 Maastricht University, Maastricht 6229 ER, The Netherlands

## Abstract

In this study, we introduce a novel encoding algorithm utilizing contrastive learning to address the substantial data size challenges inherent in mass spectrometry imaging. Our algorithm compresses MSI data into fixed-length vectors, significantly reducing storage requirements while maintaining crucial diagnostic information. Through rigorous testing on data sets, including mouse bladder cross sections and biopsies from patients with Barrett’s esophagus, we demonstrate that our method not only reduces the data size but also preserves the essential features for accurate analysis. Segmentation tasks performed on both raw and encoded images using traditional *k*-means and our proposed iterative *k*-means algorithm show that the encoded images achieve the same or even higher accuracy than the segmentation on raw images. Finally, reducing the size of images makes it possible to perform t-SNE, a technique intended for frequent use in the field to gain a deeper understanding of measured tissues. However, its application has so far been limited by computational capabilities. The algorithm’s code, written in Python, is available on our GitHub page https://github.com/kskrajny/MSI-Segmentation.

## Graphical abstract

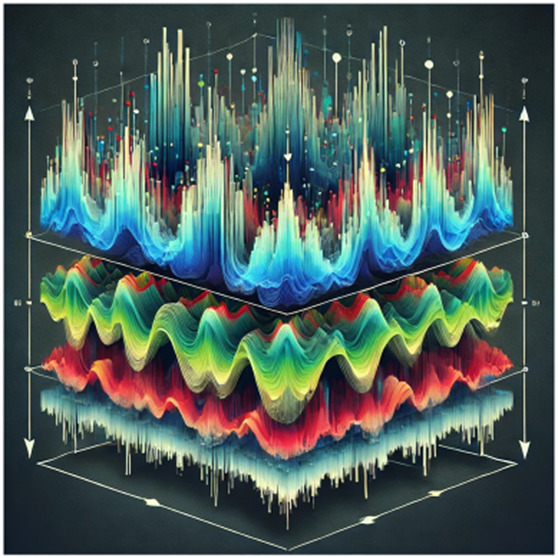

## Introduction

High-resolution mass spectrometry imaging (MSI) data are invaluable for detailed tissue analysis. They directly address the complexity of tissue biochemical diversity by capturing mass spectra from each pixel of the tissue cross-section. This technique enables precise mapping of the spatial distribution of proteins, lipids, and metabolites and provides a detailed molecular composition across varying tissue states. The development of MSI has contributed to biomarker discovery, enhancement of therapeutic methods, and understanding of disease mechanisms, demonstrating its critical role in precision medicine advancements.^1–4^

The current landscape of MSI data analysis increasingly relies on machine learning and neural networks.^5^ Sarkari et al. investigated the application of *k*-means and fuzzy *k*-means clustering to MSI data, examining the impact of preprocessing steps and parameter settings on uncovering biologically significant patterns.^6^ A self-supervised clustering approach employing contrastive learning and deep convolutional neural networks has been demonstrated to classify molecular colocalizations in MSI data, facilitating the autonomous identification of colocalized molecules.^7^ The use of convolutional neural networks was applied to enhance feature extraction and interpretability of complex biological MSI data.^8^

The mentioned tools, while powerful, are significantly challenged by the substantial size of MSI data sets. The extensive memory requirements and computational load imposed by these large volumes of data can hinder the efficiency and feasibility of employing machine learning and neural networks for in-depth analysis. To address these limitations, researchers aim to optimize the computational efficiency of data processing. For example, Alexandrov et al. introduced segmentation techniques that either uniformly select neighbors or adaptively consider neighboring spectral similarities, maintaining linear complexity and memory demands.^9^ In a similar effort to enhance computational efficiency, Dexter et al. developed a graph-based algorithm with a two-phase sampling method designed for more efficient segmentation of anatomical features and tissue types, thereby aiming to reduce the high CPU and memory usage of conventional methods.^10^

In this work, we approach the challenge of memory limitations from a different angle. Rather than merely accelerating analytical algorithms, we introduce an encoding algorithm designed to preprocess MSI data, significantly reducing its size and making it more manageable for subsequent analysis. Our algorithm utilizes contrastive learning, a method that teaches the model to distinguish between similar and dissimilar data points by comparing instances without explicit data set knowledge.^11^ This process trains the model to generate similar outputs for analogous inputs and varied outputs for dissimilar ones. Through the application of contrastive learning, our algorithm efficiently learns high-level features from MSI data, effectively compressing the data while preserving the essential information needed for analysis. Notably, the encoding process also adjusts the data’s output distribution (by setting handy means and standard deviations where required), making it easier for subsequent analytical algorithms to operate. As a result, this methodology not only streamlines data handling but also enhances the performance of analytical tools employed on the encoded data.

Our algorithm efficiently compresses each MSI pixel’s spectrum into a fixed-length vector of float numbers, regardless of its initial memory size. For instance, an image that initially occupied 1.5 GB was compressed to 8.5 MB, dramatically reducing storage requirements and enhancing the image’s manageability. Importantly, it should be noted that this transformation is reversible; the encoded spectra can be decoded to retrieve the original input spectrum, with only minor quality downgrade, yet ensuring that no critical data are lost in the compression process.

## Materials and Methods

To rigorously evaluate the performance and reliability of our encoding algorithm, we conducted our experiments using two specific data sets: a mouse bladder cross-section image and a set of images of biopsies from patients with Barrett’s esophagus (Barrett’s esophagus is a precancerous condition characterized by the abnormal transformation of esophageal cells, often due to chronic acid reflux). Both data sets were chosen since they provide reliable baseline models, which aid in assessing the correctness of our method.

### Mouse Bladder Image

The mouse bladder image was downloaded from the PRIDE database, ID PXD001283.^12^ The image is 260 × 134 pixels (34,840 pixels in total), with a pixel size of 10 μm. Further details on the sample preparation, data acquisition, and processing can be found in Römpp et al.^13^ A histological staining of the tissue section used to generate the image indicated eight distinct morphological regions.^13^ To obtain a ground truth segmentation of the MS image, used to evaluate the accuracy of our algorithm, we have generated ion images of *m*/*z* 422.93, 824.55, and 851.64 Da. We have overlaid the images to highlight regions with different chemical compositions. Using the overlaid ion images and guided by the histological staining, we have delineated different morphological regions manually in the GNU Image Manipulation Program.^14^ The resulting segmentation is shown in Supplementary Figure 7.

### Barrett’s Esophagus Biopsies Images

A data set of MS images of esophagus biopsies was downloaded from the PRIDE database (PXD028949). The data set comprises 19 images in profile mode, with sizes ranging from 1370 to 7137 pixels. Annotations provided by a trained pathologist distinguish between the epithelial tissue and stroma. Moreover, the epithelial tissue may be affected by Barrett’s esophagus, and thus, the tissue is further classified into levels of dysplasia: high-grade, low-grade, and nondysplastic (healthy tissue), which we use for aggregate patients. Using histopathological tissue labeling, we can verify whether the segmentation performed on the images encoded by our algorithm accurately differentiates epithelial tissue and stroma. More details on the data are available in the study by Beuque et al.^15^

### Encoding Algorithm

The encoding algorithm aims to reduce data size and ensures that encoded spectra possess a regular probability distribution that can be efficiently dealt with by neural networks and other analytical algorithms. We refer to the encoded spectra as *embeddings*. The size of embeddings is parameter-controlled; for instance, in the case of the mouse bladder image, we set it to a vector of length 64. The encoding operation is reversible; i.e., the information in the mass spectrum can be retrieved by decoding its embedding with only minor quality loss.

We consider each pixel of MS images as a one-dimensional intensity vector by aggregating mass spectra to *m*/*z* values rounded to the first decimal. Since our method is based on convolutional neural networks, profile-mode spectra are a more natural input than centroid mode.^16^ In particular, profile-mode spectra allow for a smoother convolution and are less prone to shifting the locations of features due to mass accuracy errors than centroid-mode spectra. Accordingly, for MS images in centroid mode, we recommend preprocessing them with a Gaussian filter to generate continuous spectra before processing them with our neural network. Our tests performed during the development of the algorithm suggest that the network can process centroid-mode spectra but with a slight decrease in accuracy of the results. For MS images in profile mode, preprocessing with a Gaussian convolution filter also has the advantage of smoothing the data and is a common step in computational MS analyses. For the Barret’s Esophagus and the Mouse Bladder data, we have applied a Gaussian convolution with σ = 0.1 Da.

We combined contrastive learning with the encoder–decoder architecture and applied several neural network layers on top of that

- linear layer—controls the portion of the neural network that executes matrix multiplication followed by bias addition;
- normalization layer—adjusts the input features to be distributed with a mean equal to 0 and standard deviation equal to 1;
- convolutional layer—improves the process of extracting spatial hierarchies of features, enabling the network to recognize patterns, textures, edges, and other local features within data;
- transposed convolutional layer—provides the same improvements as the convolutional layer but in the opposite direction, i.e., for embedding’s decoding;

with a rectified linear unit (ReLU) used as an activation function.

The process of contrastive learning begins with generating a noisy copy of each input spectrum, called *augmentation*. In our case, this noisy copy is obtained by applying small perturbations to the original spectrum, altering intensity values by up to 10% while preserving the overall spectral structure. We consider the input spectrum and its noisy copy to be similar, while any other pair is considered not similar (*positive* or *negative pair*, respectively). To learn the neural network to distinguish whether given spectra are similar, we had to involve several loss functions.

First, we used the *contrastive loss* function based on cosine similarity to determine whether a given pair is correctly classified as positive or negative. We chose this function because it effectively captures structural differences between spectra by measuring the angle between their representations, ensuring that structurally similar spectra remain close in the latent space, even when absolute intensities vary due to experimental conditions. This makes it particularly well-suited for high-dimensional MSI data. Moreover, this approach aligns with previous applications of contrastive learning in MSI, where cosine-based metrics have proven effective in preserving meaningful spectral relationships.^7^ Let us assume that we possess spectra (*z*_1_, *z*_2_, ..., *z*_2*N*_) generated in the augmentation process, where *N* is a batch sample size. Contrastive loss function can be formulated as

1 $$\mathrm{contrastive}\;\mathrm{loss}=\alpha \sum_{i,j}l_{i,j}\cdot l_{\left(z_{i},z_{j}\right)\;\mathrm{positive}\;\mathrm{pair}}$$

where

2 $$l_{i,j}=-\log\frac{\exp\left(\mathrm{sim}\left(z_{i},z_{j}\right)/\gamma \right)}{\sum_{k=1}^{2N}l_{k\neq i}\;\exp\left(\mathrm{sim}\;\left(z_{i},z_{k}\right)/\gamma \right)}$$

α and γ are constants, sim stands for the cosine similarity, and $l_{q}$ is an indicator function equal to 1 when a given condition *q* is satisfied, and 0 otherwise.

Simultaneously, we controlled if embeddings came from some sort of regular probability distribution. Let us denote by *w*_*i*_ an embedding corresponding to a spectrum *z*_*i*_. First, we use mean loss function given by

3 $$\mathrm{mean}\;\mathrm{loss}=\frac{1}{2N}\sum_{i=1}^{2N}\mu^{2}\left(w_{i}\right)$$

where μ­(*w*_*i*_) is a mean of the *i* ^th^ embedding in the batch. The aim of applying this loss function is to keep embeddings around 0. Moreover, we compute standard loss function defined as

4 $$\mathrm{standard}\;\mathrm{loss}=\frac{1}{F_{e}}\sum_{i=1}^{F_{e}}{\left(\sigma_{i}\left(w\right)-1\right)}^{2}$$

where σ_*i*_(*w*) stands for the standard deviation of the *i* ^th^ feature in the batch of embeddings and *F*_e_ is a number of the features. By *features*, we understand individual positions in embedding vectors. Using the standard loss in our neural network training process guarantees that features’ distributions will not deviate from the average value too much.

So far, each of the applied loss functions ensures that the encoder will provide embeddings that differentiate spectra correctly and are handy for the segmentation process. However, it is equally important to ensure that these embeddings can be decoded back into meaningful spectra. To achieve this, an additional loss function is required to compare the original spectrum *z*_*i*_ with its encoded and subsequently decoded counterpart *z̃*_*i*_. This type of loss is commonly termed decoder loss. In our case, we employed the mean squared error (MSE) loss function for this comparison, which is expressed as

5 $$\mathrm{MSE}\;\mathrm{loss}=\frac{1}{F_{s}}\sum_{i=1}^{F_{s}}{\left(z_{i}-{\mathrm{z̃}}_{i}\right)}^{2}$$

with *F*_s_ denoting sample vector length. Notably, continuous monitoring through decoder loss ensures that the encoded and subsequently decoded spectrum remains close to the original. We note that this approach calculates the average MSE distance between the original and decoded spectra over all pixels. Therefore, changing the intensities of high-intensity peaks present in many pixels incurs a large penalty. However, peaks with low intensities or peaks present in only a small number of pixels can be lost during the encoding.

A sketch of the encoder–decoder architecture, along with the loss functions used, is presented in Figure 1. Moreover, numerous examples of loss trajectories across training can be found in the notebooks on our GitHub page.

**Figure 1:**
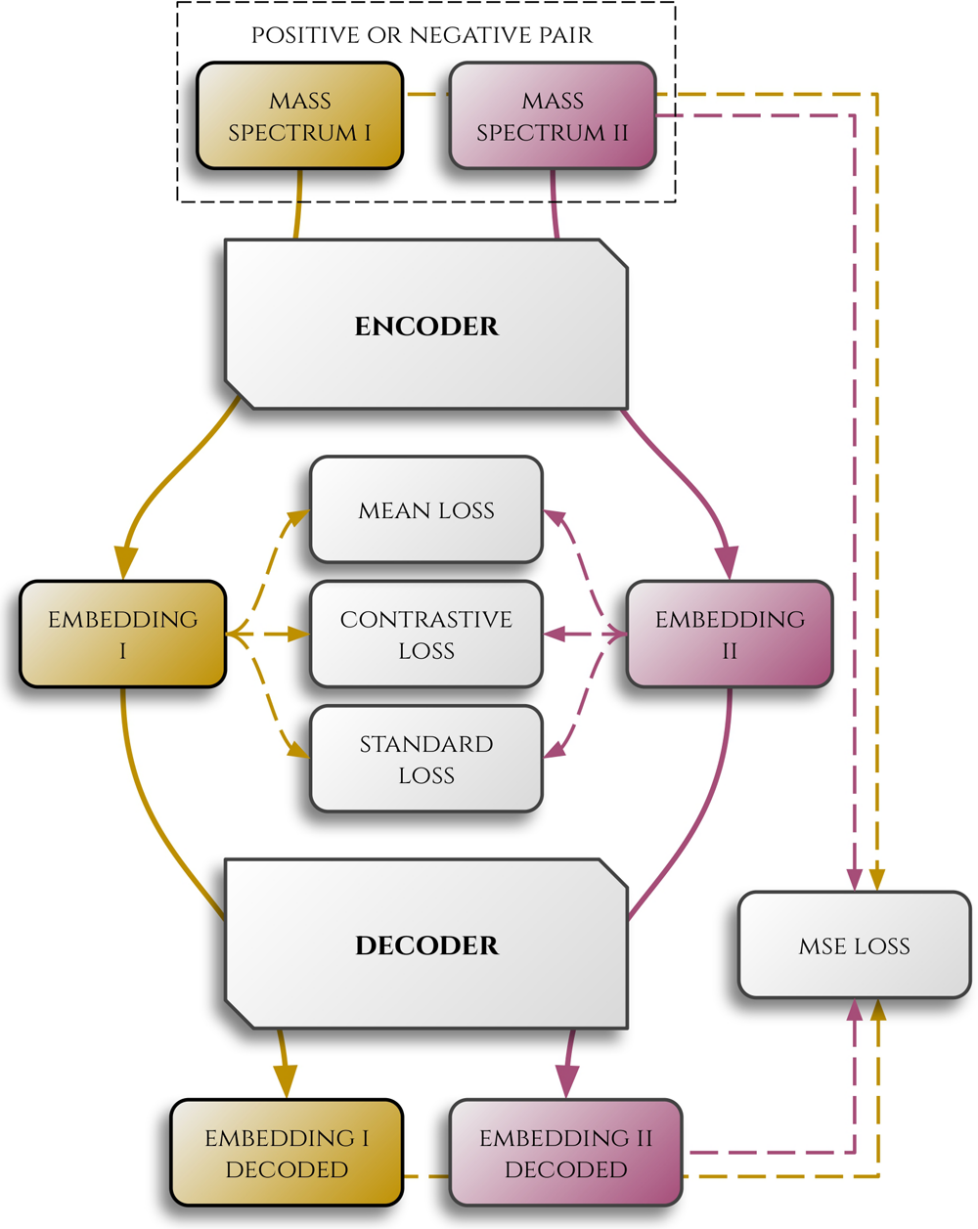
A graph illustrating the encoder–decoder learning process. First, a contrastive loss is computed to compare whether embeddings are close or distinct for the positive and negative pairs, respectively. Simultaneously, mean and standard losses are calculated to ensure that the distribution of embeddings is neat. Then, embeddings are decoded, and an MSE loss is computed to check whether the decoded and input spectra are homogeneous.

### Encoder Learning and Parameters

Optimization for each data set was achieved using the Adam optimizer, with settings of a 10^–3^ learning rate and a 10^–5^ weight decay. Losses were predominantly equalized at a weight of 1, except for the decoder loss, emphasized at 100. The batch size parameter was set at 64. If no improvement in the weighted sum of losses is noted, the patience parameter responsible for stopping training was set at 30 epochs. The weighted sums of the losses were 10^–2^ for mean and standard deviation losses and 10^–1^ for contrastive loss, with decoder loss adjustments based on data set specifics: 10^–2^ for mouse bladder and 1 for biopsies images. The computational framework was Google Colaboratory, using a Tesla T4 GPU.

### Image Segmentation

To validate whether the compression process preserves essential features, we conducted segmentation, one of the most critical tasks in analyzing MSI data, in which our encoding algorithm finds a significant application. For this purpose, we chose the *k*-means algorithm due to its two advantages: first, it is commonly used in the field, and second, it particularly benefits from the regularity in data distribution that our encoding ensures.

Note, however, that in real-world scenarios the exact number of clusters in an image is often unknown. Therefore, the *k* parameter typically needs to be chosen with a margin. However, this approach introduces another challenge: multiple clusters may represent the same tissue type and must be merged. In our case, we utilize a baseline model for testing purposes, as described in the next section. In real-world applications, a laboratory specialist must perform this merging manually. To reduce or, ideally, eliminate this necessity, we propose a modification to the traditional *k*-means algorithm, called *iterative k-means*, which serves as a practical alternative in certain scenarios.

The iterative *k*-means algorithm enhances the *k*-means clustering process by integrating it with Principal Component Analysis (PCA) to dynamically determine the optimal number of clusters based on the silhouette score^17^ for each principal component. This approach iteratively adjusts the cluster count until it meets or surpasses a predefined target. The process is outlined as follows:

**Input:** Data points D , maximal number of clusters *K*.
**Initialize:** Apply PCA to D to obtain principal components. Set the initial cluster count *k* = 1 and the currently considered principal component *i* = 1.
**Step 1:** For the *i* ^th^ principal component, execute *k*-means with varying cluster counts, identifying the optimal number *c*, based on the silhouette score. If *c* = 1, go to Step 4.
**Step 2:** Set *k* = *k* · *c* and *i* = *i* + 1.
**Step 3:** Repeat Steps 1 and 2 until *k* ≥ *K*.
**Step 4:** Aggregate the data points into final clusters by consolidating the saved clustering results.

Note that since the iterative *k*-means algorithm processes principal components sequentially, starting from the most informative ones, it typically requires only a few components to estimate the number of clusters. For instance, in the case of the mouse bladder image, it used just 6 components out of the 64-dimensional latent space. Moreover, note that as the purpose of iterative *k*-means is to accurately estimate the number of classes, applying it to images where only two or three classes are expected is unnecessary, since the results will be equivalent to those obtained with the standard *k*-means algorithm. We recommend using the iterative version for images where the number of classes is uncertain but suspected to be at least four.

Following the completion of segmentation, *convolutional smoothing* is applied to refine the results. This phase involves iteration over each pixel and executes a majority voting procedure. We assess the class assigned to the target pixel and its neighbors. The class that predominates among these is then designated as a new class for the pixel.

### Matching and Segmentation Accuracy

To assess the accuracy of the segmentation results against the actual data (ground truth or baseline model), it is necessary to align the classes identified by the segmentation algorithm with those in the baseline model. To achieve this, we use a voting procedure known as *matching*. For each class identified by the segmentation algorithm, we examine the corresponding pixels’ classes in the baseline model image. The class from the segmentation is then matched to the baseline model class that appears most frequently among those pixels. A graphical representation of this process is provided in Figure S1 in the Supporting Information. Lastly, an image with matched classes enables the verification of class alignment with the baseline model and calculation of the segmentation accuracy. Note, however, that in the MSI field baseline models are themselves acquired using specific methods. Therefore, since “accuracy” is computed relative to these baseline models, it should be interpreted more as a correlation with the results of another method rather than an absolute measure.

A visual summary of the process steps described in this section is presented as a workflow in Figure 2.

**Figure 2:**
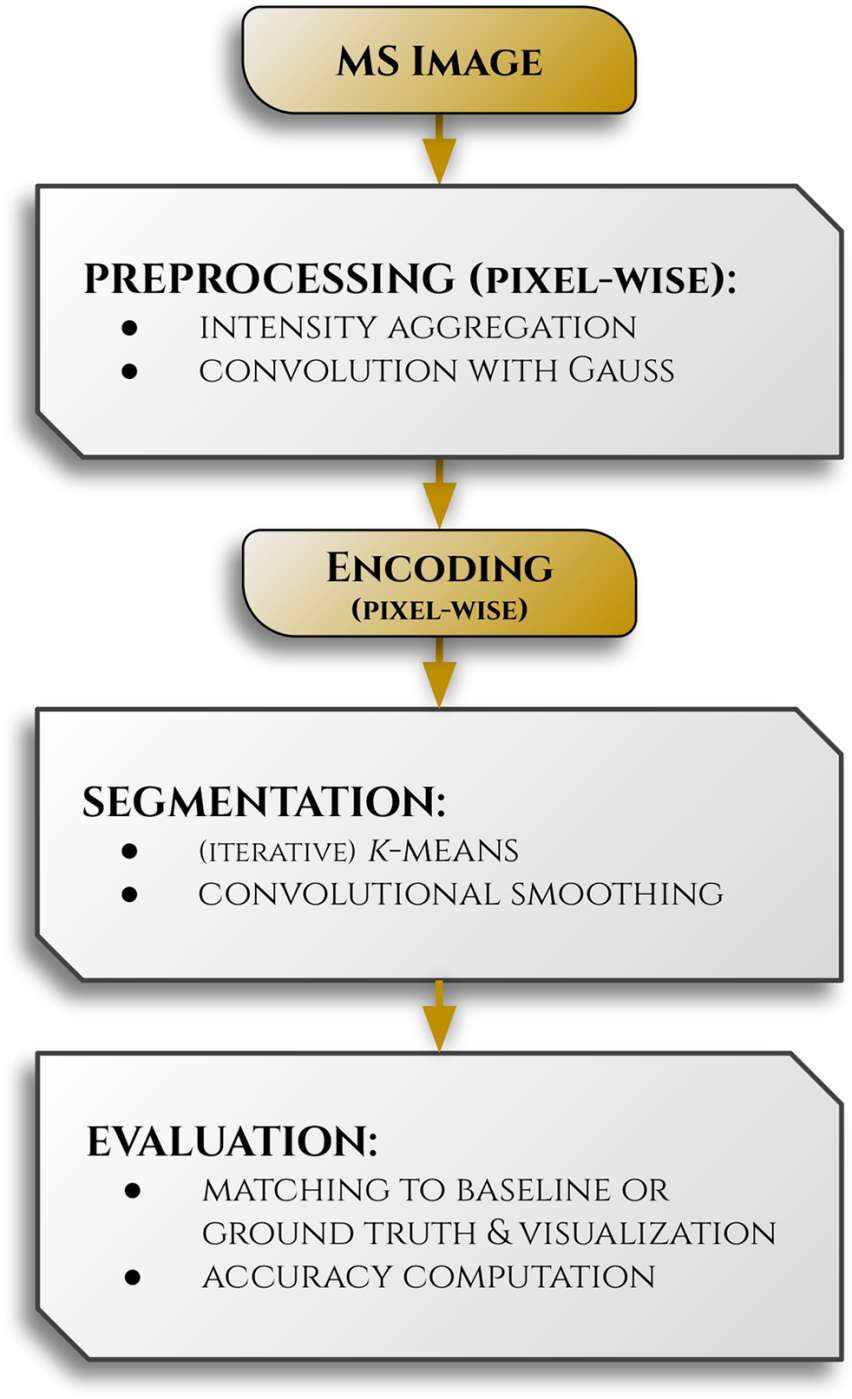
A workflow illustrating the processing of MS images, highlighting the encoding step as the core focus of our work. *Segmentation* and *Evaluation* are included primarily to demonstrate the efficacy of the encoding process. Detailed descriptions of each point are provided in the Methods section.

### Algorithm Availability

The computations were performed in Python, primarily using the PyTorch^18^ and scikit-learn^19^ packages. The entire code, including neural network training and segmentation implementations, is freely available under the MIT license on our GitHub page: https://github.com/kskrajny/MSI-Segmentation. Additionally, our GitHub page provides detailed parameters and plots of individual loss functions recorded during neural network training for each data set.

## Results and Discussion

### Encoding Algorithm’s Performance

In the first data set, consisting of the mouse bladder image, our goal was to compare segmentation performance on raw and encoded data. The image, stored in the Apache Parquet format, had an initial size of approximately 1.5 GB. The encoder’s training process, which varied between 1.5 to 2.5 h depending on the parameters, successfully compressed each image pixel’s spectrum into a 64-element vector. Here, it is critical to note that the reported computational times refer to training the encoder, not the encoding process itself. Once the model is trained, encoding and decoding of entire MS images take only a few seconds. This compression reduced the file size to 8.5 MB, achieving a 99.4% reduction in memory usage.

Next, we applied the t-SNE algorithm to the image. This algorithm is typically used for the preliminary analysis of MS images to gain a deeper understanding of the measured data and to estimate the number of biochemical classes that can be expected in the analyzed images. However, the high computational demands of t-SNE often make it challenging to apply it to raw images. In contrast, its application to encoded images proved successful. Since we had ground truth data and our primary goal was to demonstrate the feasibility of t-SNE on encoded images, we used scikit-learn’s default hyperparameters, except for the perplexity parameter, which was set to 10^3^, without further optimization. Notably, the t-SNE analysis on the encoded image was completed in approximately 50 min using sckit-learn’s default hyperparameters. A selected dimension of the t-SNE output is presented in Figure 3, indicating that at least three distinct biochemical categories can be expected. Additional examples can be found in the Supporting Information.

**Figure 3:**
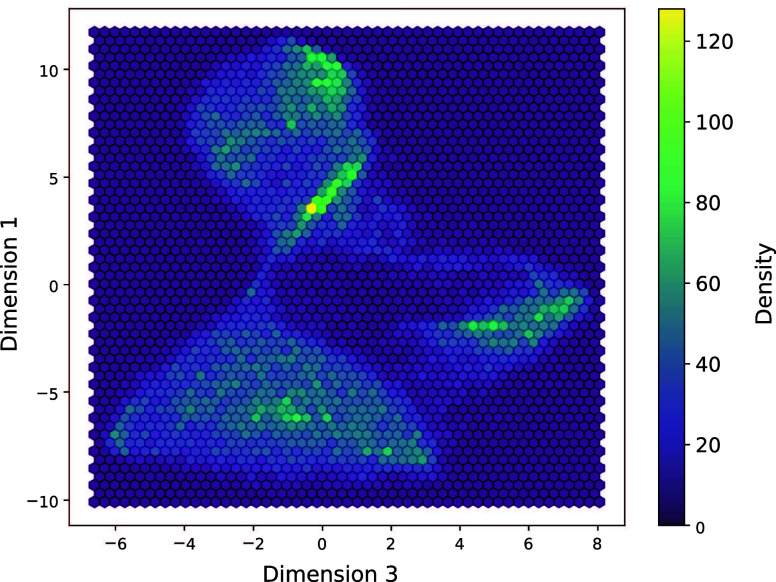
Illustration of the t-SNE algorithm output computed on the encoded image of the mouse bladder. Selected dimensions are showcased, demonstrating the minimal number of three biochemical classes can be expected from segmentation. The computations were performed using scikit-learn with a perplexity parameter of 10^3^, while all other hyperparameters remained at their default settings with no further optimization. The process was completed in approximately 50 min.

Finally, we conducted a segmentation task to verify that the encoding process did not lose any essential information. The *k*-means algorithm was applied to both raw and encoded images, whereas the iterative *k*-means algorithm was used exclusively on encoded images due to the high computational demands of applying it directly to raw data. Additionally, we evaluated clustering algorithms on the highest peaks from the spectrum by calculating the mean for each spectral index across the image and selecting the 128 indices with the highest means. Segmentation accuracies are detailed in Table 1, and their visualizations are presented in Figure 4. More segmentations, using different parameters and an alternative baseline model, are available in the Supporting Information.

**Figure 4:**
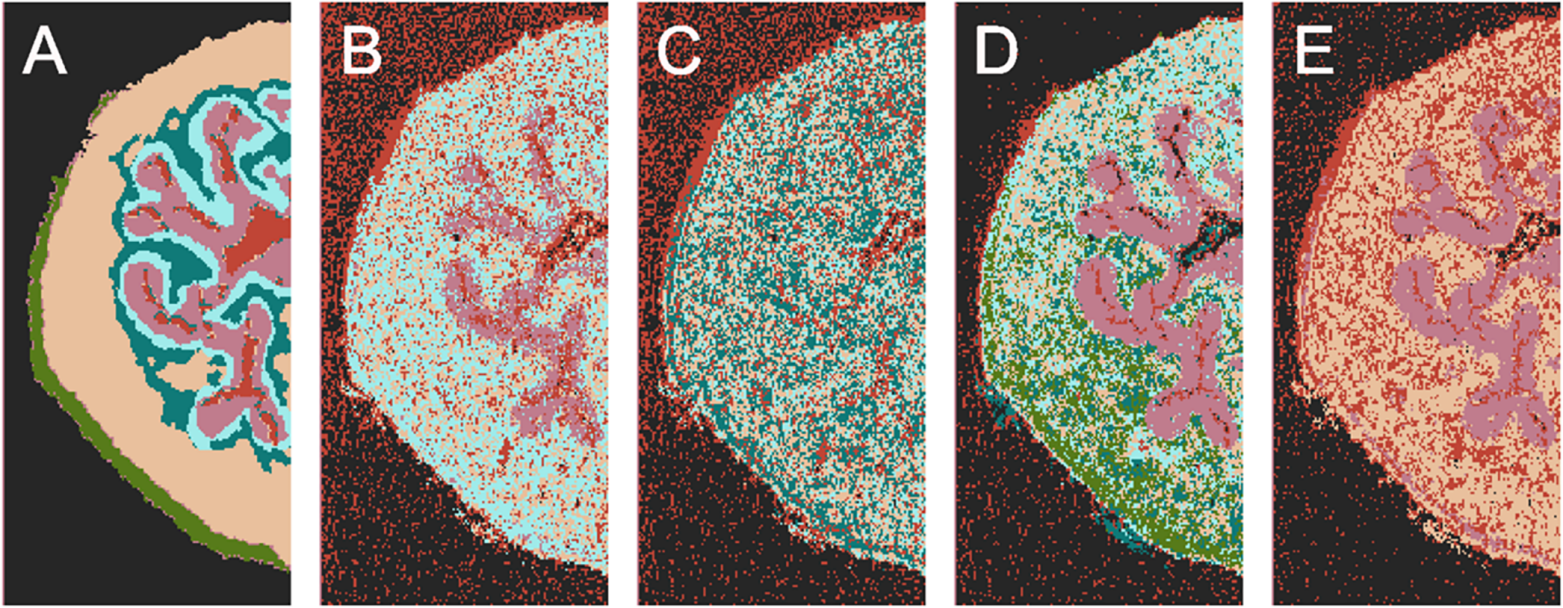
Segmentation results for the mouse urinary bladder MS image with the matching procedure were applied. Panel (A) shows the baseline model, as described in the Mouse Bladder Image section. The following panels present segmentation results: (B) *k*-means on the original image, (C) *k*-means on the 128 highest peaks of the original image, (D) *k*-means on the encoded image, and (E) iterative *k*-means on the encoded image. While the ground truth model consists of seven clusters, we did not use this knowledge directly; instead, we selected *k* = 12 to allow for a margin. In the case of iterative *k*-means, the number of clusters was estimated autonomously, yielding eight clusters. These were then matched to the baseline model to enable segmentation accuracy assessment and clearer visualization, resulting in 5, 6, 7, and 4 classes for panels B–E, respectively. Segmentation accuracies are reported in Table 1, and segmentations without the matching procedure applied are provided in the Supporting Information.

**Table 1:** Accuracies of Segmentation Tasks into Seven Classes for the Mouse bladder Image Using the *k*-means Algorithm^a^

method	data	accuracy (%)
*k*-means	raw image	42.67
*k*-means	top-128	41.28
*k*-means	encoded image	59.01
iterative *k*-means	encoded image	**64.44**

a Segmentation was performed on raw and encoded images and additionally on the highest 128 peaks of each pixel’s spectrum in the raw image (denoted by top-128). Moreover, the iterative *k*-means was applied to the encoded image. Note that accuracy computation was possible by the availability of a baseline model, which is often not a real-life scenario.

In contrast to the mouse bladder image, the Barrett’s esophagus biopsy images are much larger, a size commonly encountered in mass spectrometry imaging. This allowed us to demonstrate the encoder algorithm’s performance on significantly larger data sets in terms of individual image size and patient count. Consequently, we encountered a challenge our work aims to address: computing segmentation on raw images was not feasible due to extensive CPU requirements. Therefore, as a basic solution, we once again employed the commonly used naive approach that involves applying a segmentation algorithm only to the highest peaks in the pixels’ spectra, with the number of peaks fixed at 128.

The complete data set is 54.21 GB in size. Training the encoder to compress the data into 128-length vectors for the entire data set took approximately 2 h, resulting in a compressed size of 81.26 MB—representing a 99.85% reduction in memory usage. Each patient’s images were encoded individually, enabling the encoder to focus on patient-specific tissue variations rather than interpatient differences. Segmentations were performed to distinguish tissue types: epithelial tissue and stroma. The segmentation results are summarized in Table 2, where cross-section images are grouped according to the patients’ dysplasia classifications. Detailed results for each individual cross-section, along with exemplary visualizations of the segmentations, are available in the Supporting Information.

**Table 2:** Summary of the Compression Process and Tissue Segmentation Results, Grouped by Barrett’s Esophagus Overall Classification^a^

	images’ storage size		computation	tissue type seg. acc. (%)	
patients’ classification	raw (GB)	encoded (MB)	time (min)	encoded image	top-128
nondysplastic	11.59	17.37	26.08	71.33	66.39
low-grade dysplasia; nonprogressive	16.03	24.05	36.10	70.42	65.24
low-grade dysplasia; progressive	15.69	23.51	35.28	68.02	65.18
high-grade dysplasia	10.90	16.33	24.52	66.87	62.12
	Σ = 54.21	Σ = 81.26	Σ = 121.98	avg. = 69.16	avg. = 64.73

a The left side of the table details the compression process, including data size before and after encoding and encoder training times. The right side presents segmentation outcomes, distinguishing tissue types: epithelial tissue and stroma. Segmentation was performed using the k-means algorithm, with the 128 highest peaks (“top-128”) used as a baseline due to the high computational demands of applying *k*-means directly to raw images.

### Storage of MS Images

Finally, we highlight one more application of our encoding algorithm: storage of MS images. Storing all acquired data can be a significant challenge for laboratories conducting extensive mass spectrometry imaging. By using our algorithm, only the encoded images and their corresponding trained models, which are not memory-intensive, need to be stored. Furthermore, due to the encoder–decoder architecture, the encoded MSI images can be easily decoded whenever needed. Moreover, let us recall that the reported computational times refer to training the encoder, not the encoding process itself; once trained, encoding and decoding MS images take only a few seconds.

## Conclusions

In conclusion, we have introduced a highly efficient compression algorithm based on an encoder–decoder neural network architecture. As demonstrated, applying the encoder to an MS image significantly reduces its memory footprint and enables the execution of computations that would be CPU-intensive for raw images. For instance, the t-SNE algorithm, ideal for primary analysis for a deeper understanding of data structures, is often challenging to use on raw images, yet may be easily applied to encoded ones. Most importantly, encoding allows for segmentation tasks to be performed on images without concern about memory requirements. Additionally, segmentation algorithms like *k*-means benefit from the regular distribution ensured by the encoder, resulting in higher accuracy compared to segmentation performed on raw images, as demonstrated in our manuscript.

## Supplementary Material



## Notes

Examples of the matching procedure; comparisons between original and encoded spectra; loss function trajectories during training; optical image of the mouse urinary bladder; remaining dimension pairs from the t-SNE visualization; detailed segmentation results for individual slices of Barrett’s esophagus tissue along with exemplary images; and segmentation of the mouse urinary bladder based on an alternative ground truth model (PDF)
