# Supplementary material for: Efficient Compression of Mass Spectrometry Images via Contrastive Learning-Based Encoding
Source: Anal Chem. 2025 Jul 21;97(29):15579–85. doi: 10.1021/acs.analchem.4c06913 (PMC12311889; doi:10.1021/acs.analchem.4c06913)
Supplement: Supplementary file 1 [file ac4c06913_si_001.pdf]

Supporting Information
Efficient compression of mass spectrometry images via contrastive learning-based encoding

Piotr Radziński,[†,*] Jakub Skrajny,[†,*] Maurycy Moczulski,[†] Michał A. Ciach,[‡] Dirk Valkenborg,[¶] Benjamin Balluff,[§] and Anna Gambin[†]

† *Institute of Informatics, University of Warsaw, 00-927 Warsaw, Poland*
‡ *Department of Applied Biomedical Science, Faculty of Health Sciences, University of Malta, Msida, MSD 2080, Malta*
¶ *Interuniversity Institute of Biostatistics and Statistical Bioinformatics, Hasselt University, BE3500 Hasselt, Belgium*
§ *The Maastricht MultiModal Molecular Imaging (M4I) Institute, Maastricht University, 6229 ER Maastricht, The Netherlands*
* These authors have contributed equally to this work and share first authorship.

e-mail: pmradzinski@mimuw.edu.pl

Supporting Information Available

Table of Contents

1 Illustration of the Matching Procedure S2
2 Figure 4 without the matching procedure applied S3
3 Exemplary encoded and subsequent decoded mass spectra S4
4 An example of loss trajectories during encoder training S5
5 Complementary perspectives of t-SNE on remaining dimensions S6
6 Segmentation results on cross-sections from Barrett's esophagus patients S7
7 Model of ground truth segmentation of the mouse bladder MS image S9
8 Segmentation of mouse bladder image on alternative ground truth model S10

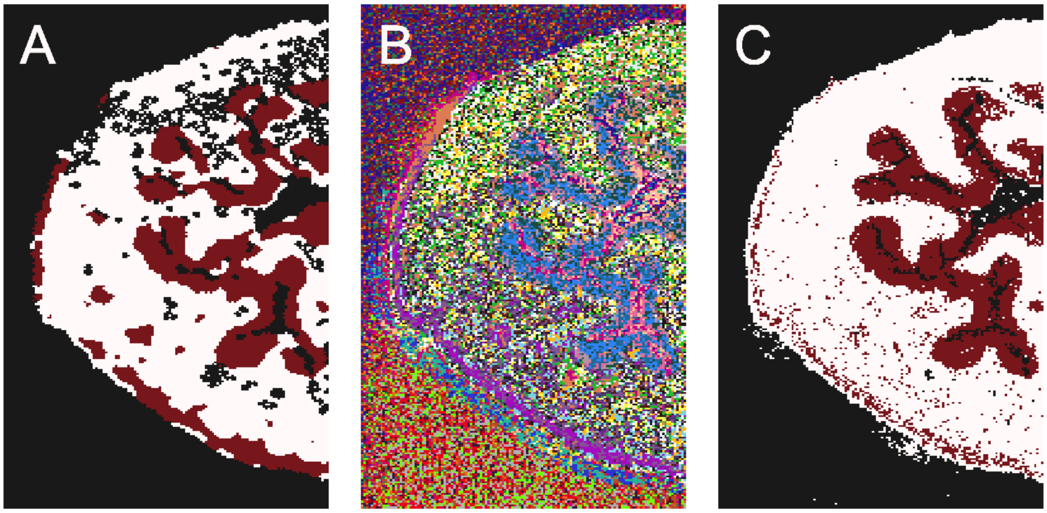

Figure S1: Illustration of the Matching Procedure. Graphical representation of the matching procedure. Panel (A) displays a baseline model data, illustrating the actual class distribution. Panel (B) shows the initial classes as identified by the k-means segmentation algorithm. Panel (C) presents the results after applying the matching procedure, aligning the algorithm's classes with those of the baseline model to facilitate accuracy assessment. This matching process involves a majority voting procedure where each class identified by the algorithm is aligned with the most frequently corresponding class in the baseline model, as described in the Matching and Segmentation Accuracy section.

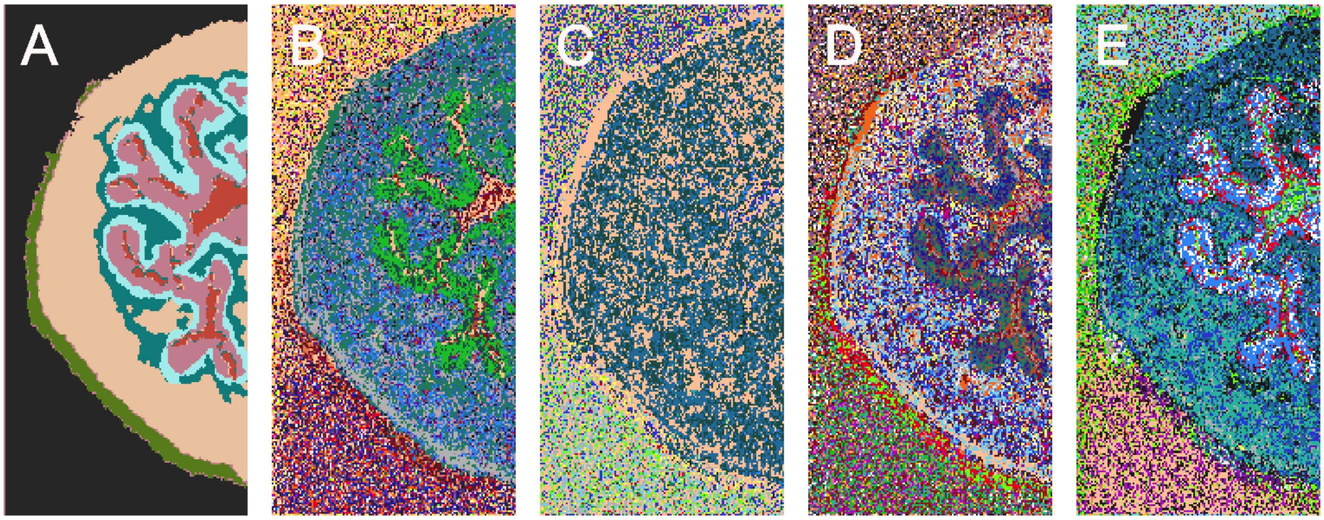

Figure S2: Segmentation results for the mouse urinary bladder MS image, as in Fig. 4, yet without the matching procedure applied. Panel (A) shows the baseline model, as described in the Mouse Bladder Image section. The following panels present segmentation results: (B) k-means on the original image, (C) k-means on the 128 highest peaks of the original image, (D) k-means on the encoded image, and (E) iterative k-means on the encoded image. While the ground truth model consists of 7 clusters, we did not use this knowledge directly; instead, we selected $k = 12$ to allow for a margin. In the case of iterative k-means, the number of clusters was estimated autonomously, yielding 8 clusters.

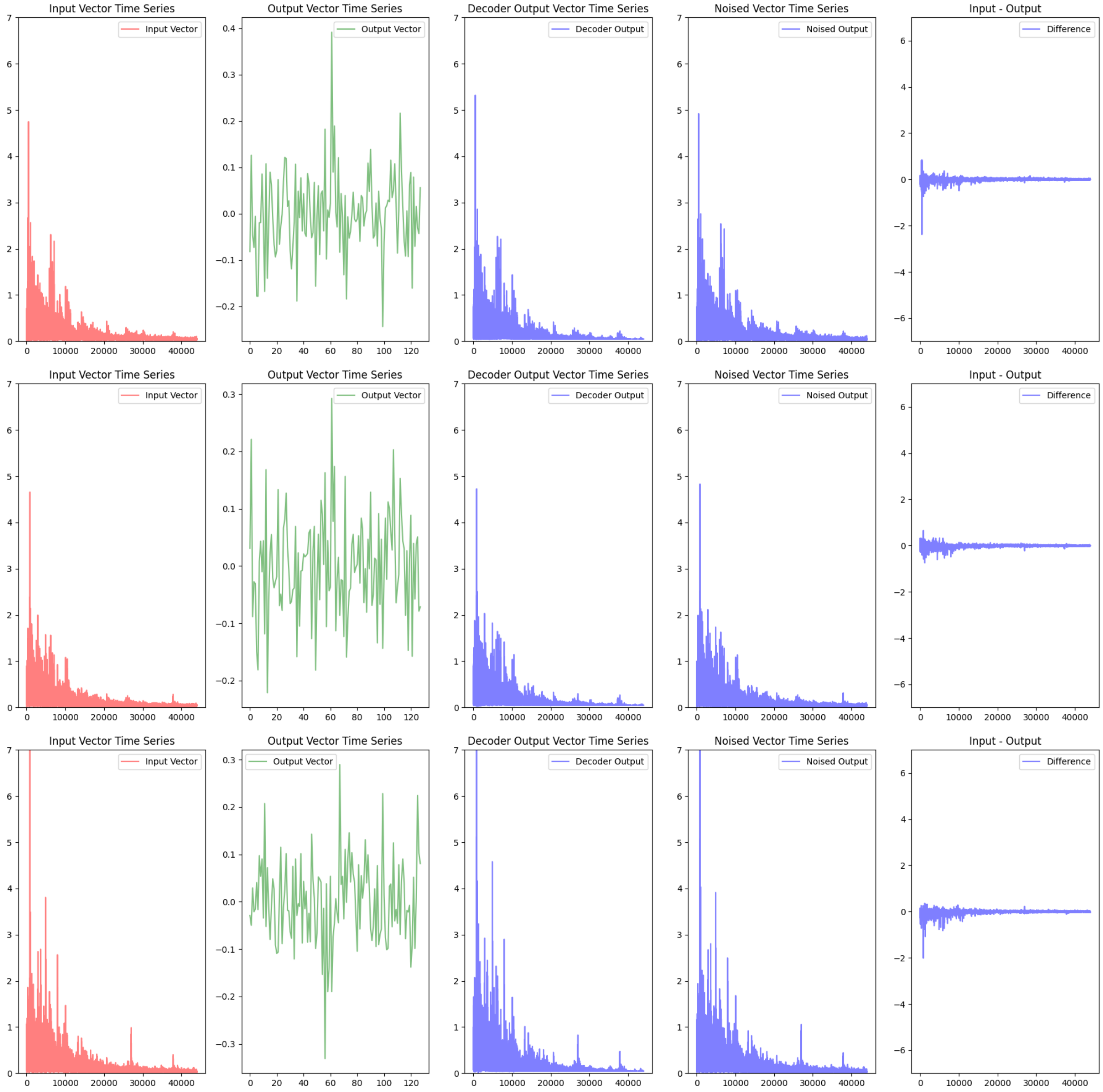

Figure S3: Three exemplary spectra illustrate how encoding and subsequent decoding affect the mass spectra. The leftmost *Input Vector* represents the original mass spectrum. The *Output Vector* shows the spectrum's embedding, i.e., its encoded representation in latent space. The *Decoder Output* displays the reconstructed spectrum after encoding and subsequent decoding. The *Noised Vector* plot shows the original spectrum with Gaussian noise added. Finally, the rightmost panel, *Input – Output*, shows the difference between the spectra in the first and third panels.

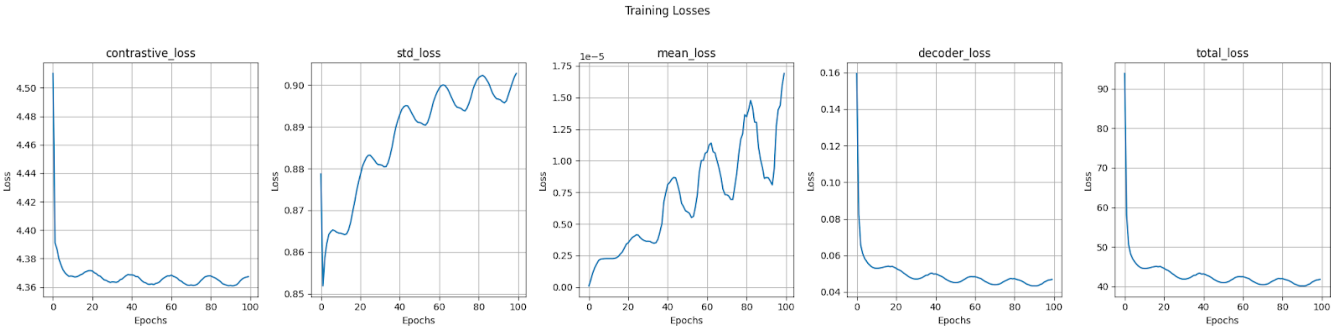

Figure S4: An example of loss trajectories during encoder training. Note that the increase in the standard and mean losses (shown in panels 2 and 3, respectively) is not a cause for concern. These are supporting losses designed to encourage the encoded spectra to adopt more “convenient” distributions. Their growth is often a natural consequence of prioritizing contrastive and decoder losses. Our primary objective is to achieve accurate encoding and decoding, while promoting a well-structured latent space remains a secondary goal. More examples can be found on our GitHub page.

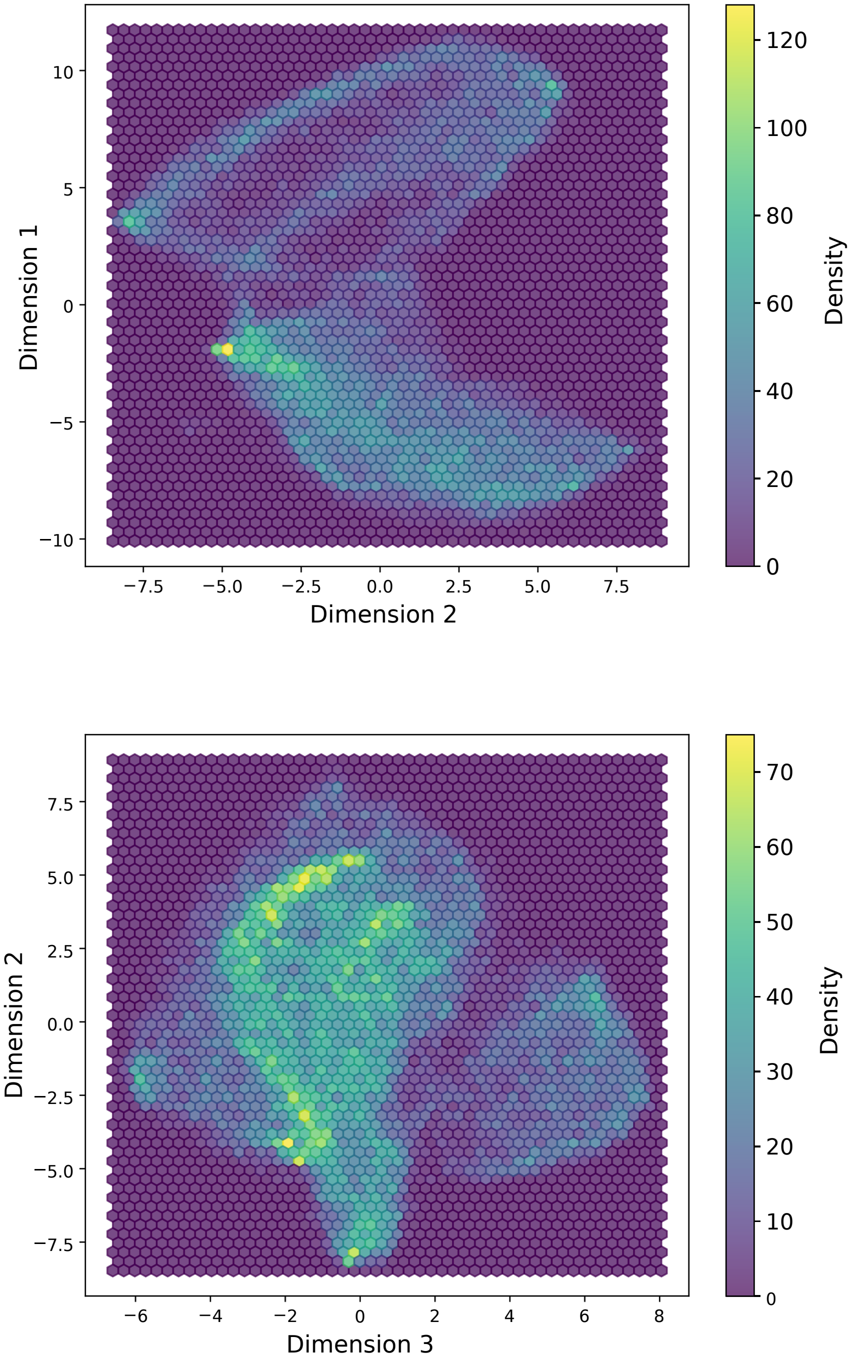

Figure S5: The two remaining perspectives on t-SNE results complement the example shown in Figure 3 for the mouse bladder image. As a reminder, the computations were performed using scikit-learn with a perplexity parameter of 10^3, while all other hyperparameters remained at their default settings, with no further optimization. The process was completed in approximately 50 minutes.

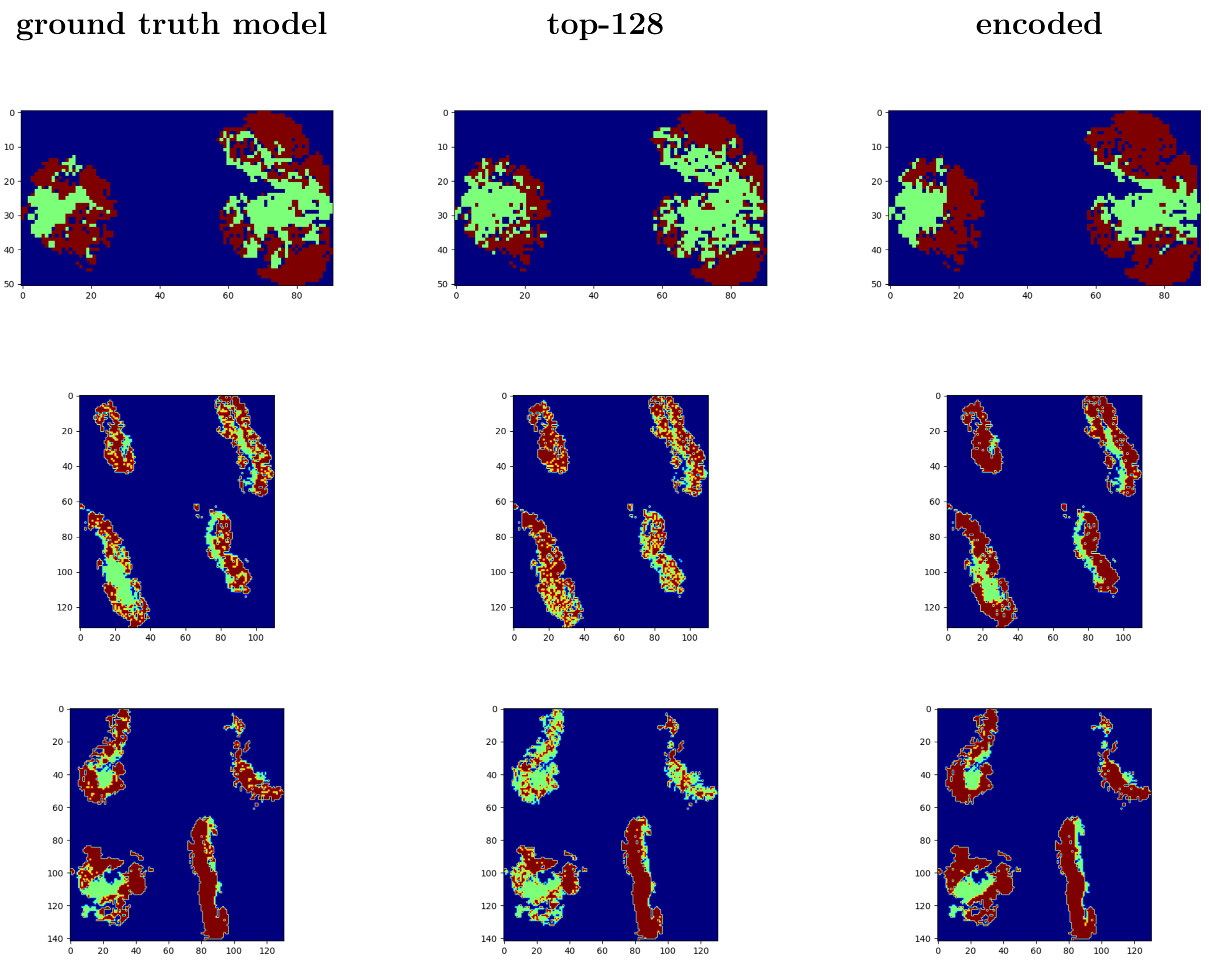

Figure S6: Exemplary segmentation results on cross-sections from Barrett's esophagus patients. The first column presents the ground truth model. The second shows segmentation results using the k-means algorithm applied to the 128 highest peaks of the original images. The third displays the results of k-means applied to the encoded images. The rows correspond to patients H2, L3, and P2, for whom detailed results are provided in Table S1.

Table S1: Detailed encoding and Barrett's esophagus images tissue segmentation.

	images' storage size		computation	tissue type seg. acc. (%)	
	raw (GB)	encoded (MB)	time (min)	encoded image	top-128
A1	0.59	0.89	1.34	63.39	59.76
A2	0.93	1.40	2.10	72.33	63.02
B2	0.43	0.64	0.96	78.01	60.68
C3	1.13	1.70	2.55	73.75	74.50
E1	0.61	0.91	1.37	63.55	57.37
F2	0.55	0.82	1.23	69.94	62.04
I1	0.62	0.93	1.40	67.27	63.49
J2	1.07	1.60	2.40	74.48	72.25
K3	1.07	1.60	2.40	63.57	62.48
M1	0.55	0.83	1.25	77.49	65.68
N2	0.54	0.81	1.22	83.69	79.53
O3	1.74	2.61	3.91	72.79	71.07
Q1	0.87	1.30	1.95	74.94	74.10
R2	0.55	0.82	1.23	63.34	59.78
S3	0.34	0.51	0.77	71.44	70.16
non-dysplastic	$\Sigma = 11.59$	$\Sigma = 17.37$	$\Sigma = 26.08$	avg. = 71.33	avg. = 66.39
A3	0.63	0.94	1.41	73.65	64.67
C1	1.20	1.80	2.70	74.91	73.66
D2	1.13	1.70	2.55	71.95	68.08
E3	0.31	0.47	0.71	78.53	77.78
H2	0.97	1.45	2.18	81.78	74.07
I3	0.93	1.40	2.10	68.20	54.19
K1	0.80	1.20	1.80	57.32	56.34
L2	2.34	3.51	5.26	80.01	75.78
M3	1.93	2.90	4.35	63.44	67.19
O1	1.81	2.71	4.07	67.48	61.43
P2	2.12	3.18	4.77	88.88	64.93
Q3	0.62	0.93	1.40	61.08	63.16
S1	0.47	0.71	1.07	62.24	54.92
T2	0.77	1.15	1.73	56.39	57.20
low-grade dysplasia; non-progressive	$\Sigma = 16.03$	$\Sigma = 24.05$	$\Sigma = 36.10$	avg. = 70.42	avg. = 65.24
B1	0.49	0.74	1.11	78.91	77.18
C2	0.58	0.87	1.31	71.96	66.98
D3	0.57	0.85	1.28	67.82	62.27
F1	0.31	0.46	0.69	61.78	65.89
H3	0.45	0.67	1.01	58.97	60.05
J1	1.00	1.50	2.25	74.24	74.01
K2	0.87	1.30	1.95	60.16	60.00
L3	1.65	2.47	3.71	77.09	59.51
N1	2.27	3.40	5.10	71.16	60.57
O2	1.58	2.37	3.55	68.17	70.24
P3	2.32	3.48	5.22	66.43	70.54
R1	1.20	1.80	2.70	67.06	64.45
S2	1.00	1.50	2.25	67.05	64.75
T3	1.40	2.10	3.15	61.51	56.01
low-grade dysplasia; progressive	$\Sigma = 15.69$	$\Sigma = 23.51$	$\Sigma = 35.28$	avg. = 68.02	avg. = 65.18
D1	0.30	0.45	0.68	59.44	61.80
E2	1.07	1.60	2.40	63.88	62.08
F3	0.80	1.20	1.80	89.86	75.98
H1	1.76	2.64	3.96	72.47	62.72
I2	0.63	0.95	1.43	64.74	58.27
J3	0.27	0.41	0.62	63.69	60.15
L1	0.93	1.39	2.09	61.99	54.28
M2	1.27	1.90	2.85	60.65	63.63
N3	0.44	0.66	0.99	62.24	52.84
P1	0.72	1.08	1.62	80.68	71.90
Q2	0.87	1.30	1.95	57.52	58.57
R3	1.07	1.60	2.40	71.17	65.06
T1	0.77	1.15	1.73	60.94	60.29
high-grade dysplasia	$\Sigma = 10.90$	$\Sigma = 16.33$	$\Sigma = 24.52$	avg. = 66.87	avg. = 62.12

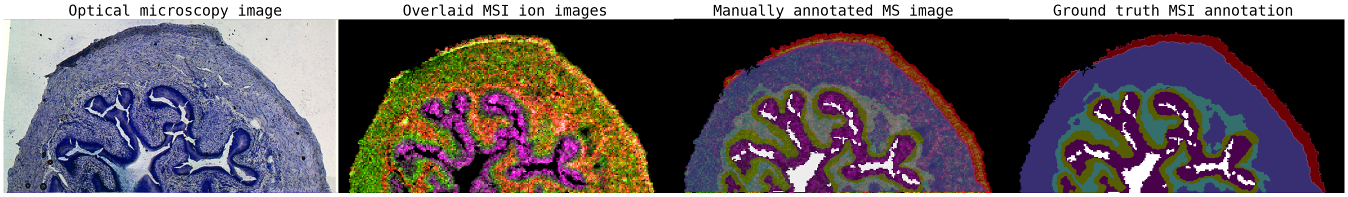

Figure S7: A ground truth segmentation of the mouse bladder MS image used to evaluate the accuracy of our algorithm. From left to right: a histological staining of the section used to generate the MS image; overlaid MS images of m/z 422.93 Da (green), 824.55 Da (magenta) and 851.64 Da (red); overlaid MS images with manually delineated segments; the ground truth segmentation represented as a 260x134 pixel image.

Table S2: Lipid ions annotated in the mouse urinary bladder image using an alternative methodology, which is applied in the segmentations in Supporting Information.

	chemical formula	M_{mono}
[PC(36:4) + K]+	$C_{44}H_{80}NO_8PK$	820.53
[PC(36:2) + K]+	$C_{44}H_{84}NO_8PK$	824.56
[PC(36:1) + K]+	$C_{44}H_{86}NO_8PK$	826.57

Complete Segmentation on Mouse Bladder Image (alternative ground truth model)

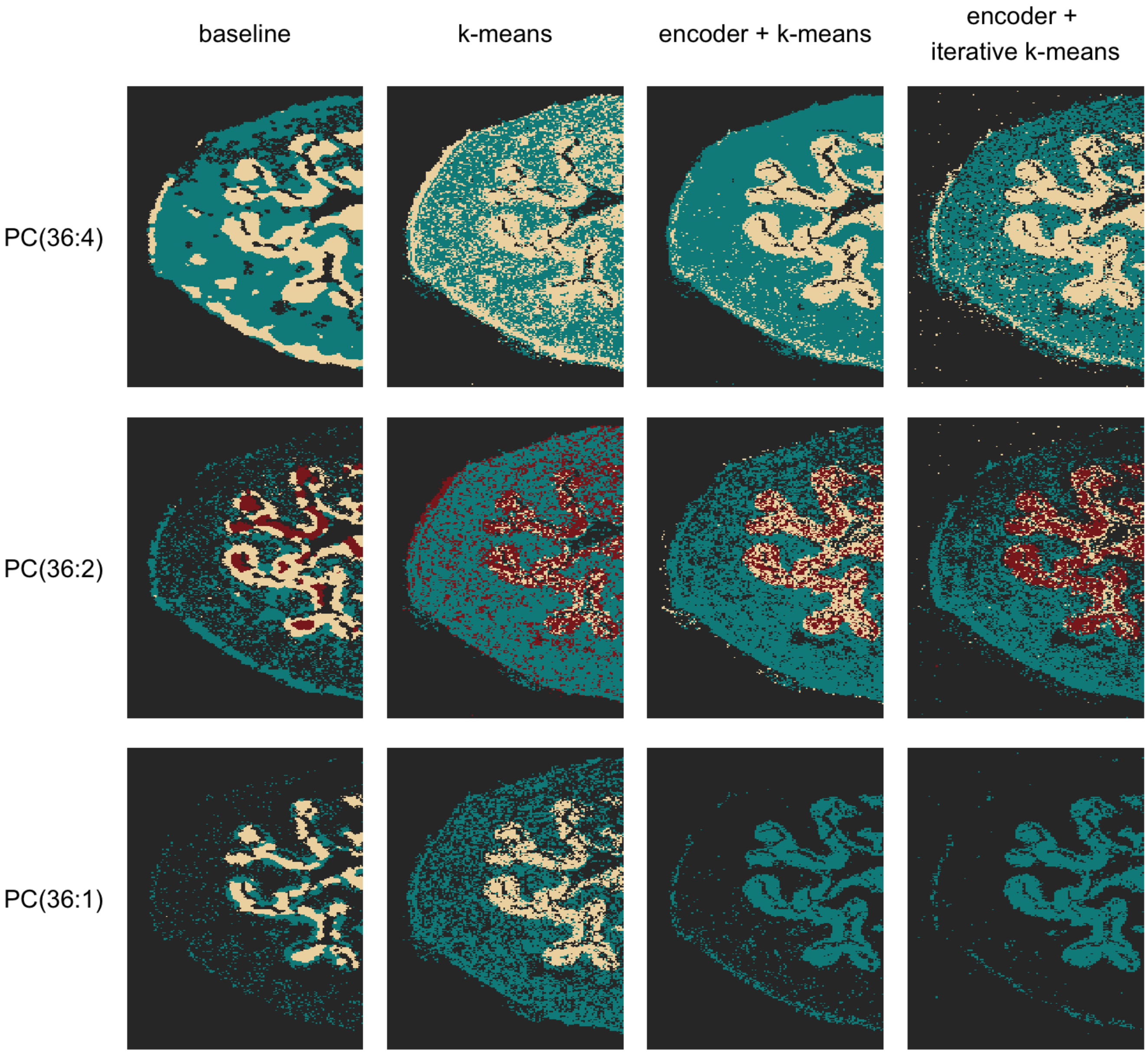

Figure S8: Segmentation of mouse bladder image for three different lipids – PC(36:4), PC(36:2), and PC(36:1), as in Table 2. The first column displays baseline model levels as described in the Methods section. Subsequent panels show segmentation results using k-means on the raw image, k-means on the encoded image, and iterative k-means on the encoded image. A matching procedure has been applied to each segmentation result for clearer visualization.

encoding	✗	✓	✓
k-means	standard	standard	iterative
PC(36:4)	77.2	**83.3**	80.3
PC(36:2)	56.3	71.3	**75.6**
PC(36:1)	70.9	86.1	**87.1**

Segmentation on Mouse Bladder Image with Alternative Parameters and Without Convolutional Smoothing (alternative ground truth model)

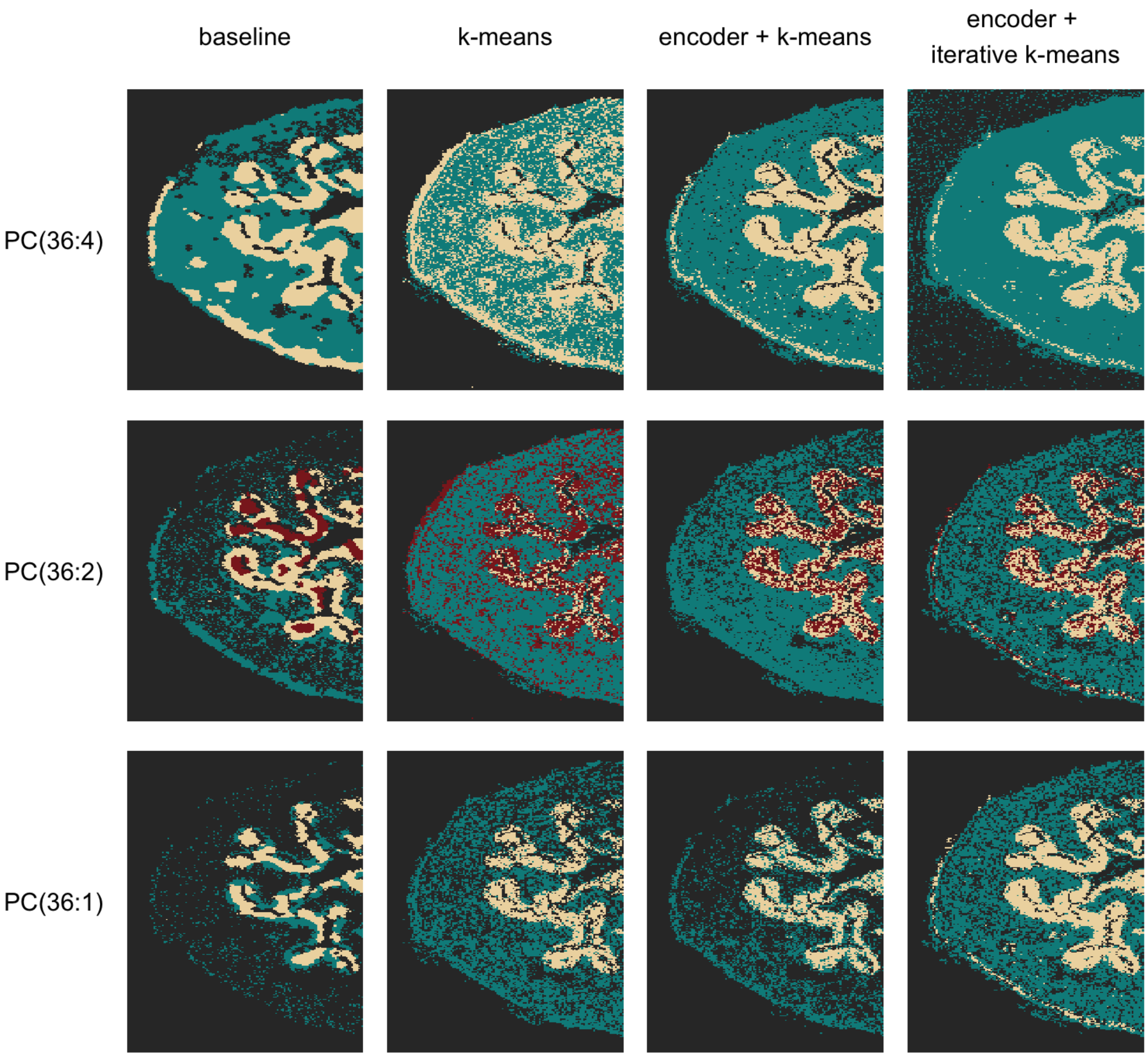

Figure S9: Segmentation of mouse bladder image as in Figure S8, but with the encoder trained on an alternative set of parameters. Additionally, no smoothing procedure was applied.

encoding	✗	✓	✓
k-means	standard	standard	iterative
PC(36:4)	77.2	**84.2**	77.1
PC(36:2)	56.3	**70.3**	67.8
PC(36:1)	70.9	**82.9**	63.4

Segmentation of Mouse Bladder Image with Alternative Parameters, Without Gaussian Splitting or Convolutional Smoothing (alternative ground truth model)

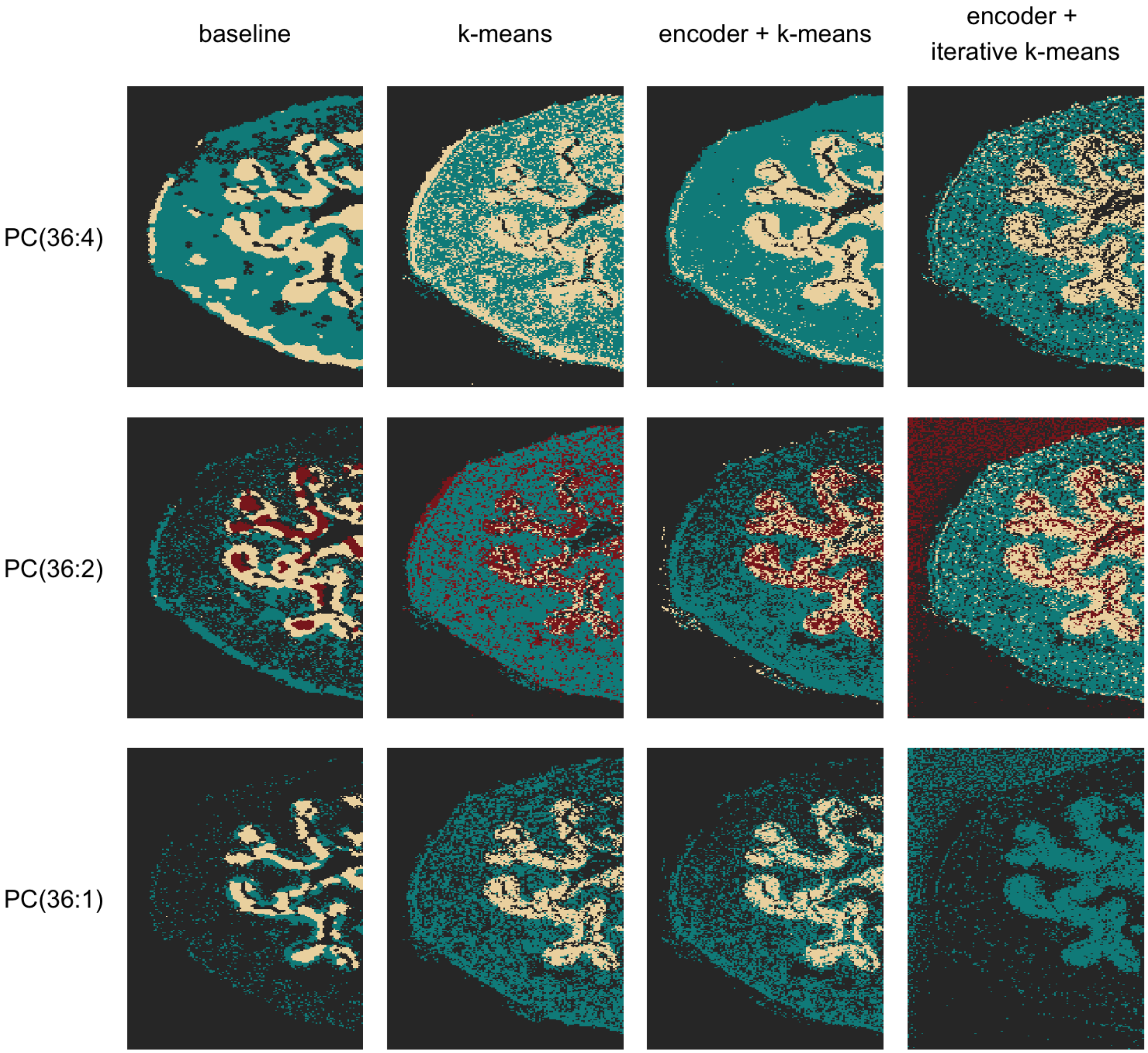

Figure S10: Segmentation of mouse bladder image as in Figure S8, but with the encoder trained on an alternative set of parameters. Additionally, the data were not preprocessed by splitting with a Gaussian distribution, and no smoothing procedure was applied.

encoding	✗	✓	✓
k-means	standard	standard	iterative
PC(36:4)	77.2	**83.3**	74.8
PC(36:2)	56.3	**74.7**	57.0
PC(36:1)	70.9	**76.9**	76.0
